# Allele specific expression and methylation in the bumblebee, *Bombus terrestris*

**DOI:** 10.7717/peerj.3798

**Published:** 2017-09-13

**Authors:** Zoë Lonsdale, Kate Lee, Maria Kiriakidu, Harindra Amarasinghe, Despina Nathanael, Catherine J. O’Connor, Eamonn B. Mallon

**Affiliations:** 1 Department of Genetics and Genome Biology, University of Leicester, Leicester, United Kingdom; 2 Bioinformatics and Biostatistics Support Hub (B/BASH), University of Leicester, Leicester, United Kingdom; 3 Academic Unit of Cancer Sciences, University of Southampton, Southampton, United Kingdom; 4 School of Biosciences, Cardiff University, Cardiff, United Kingdom

**Keywords:** Methylation, Hymenoptera, Genomic imprinting, Allele specific expression

## Abstract

The social hymenoptera are emerging as models for epigenetics. DNA methylation, the addition of a methyl group, is a common epigenetic marker. In mammals and flowering plants methylation affects allele specific expression. There is contradictory evidence for the role of methylation on allele specific expression in social insects. The aim of this paper is to investigate allele specific expression and monoallelic methylation in the bumblebee, Bombus terrestris. We found nineteen genes that were both monoallelically methylated and monoallelically expressed in a single bee. Fourteen of these genes express the hypermethylated allele, while the other five express the hypomethylated allele. We also searched for allele specific expression in twenty-nine published RNA-seq libraries. We found 555 loci with allele-specific expression. We discuss our results with reference to the functional role of methylation in gene expression in insects and in the as yet unquantified role of genetic cis effects in insect allele specific methylation and expression.

## Introduction

Epigenetics is the study of heritable changes in gene expression that do not involve changes to the underlying DNA sequence (Goldberg, Allis & Bernstein, 2007). Social hymenoptera (ants, bees, and wasps) are important emerging models for epigenetics (Glastad et al., 2011; Weiner & Toth, 2012; Welch & Lister, 2014; Yan et al., 2014). This is due to theoretical predictions for a role for an epigenetic phenomenon, genomic imprinting (parent of origin allele specific expression), in their social organisation (Queller, 2003), the recent discovery of parent-of-origin allele specific expression in honeybees (Galbraith et al., 2016), and data showing a fundamental role in social insect biology for DNA methylation, an epigenetic marker (Chittka, Wurm & Chittka, 2012).

In mammals and flowering plants, allele specific expression is often associated with methylation marks passed from parents to offspring (Reik & Walter, 2001). However DNA methylation is involved in numerous other cellular processes (Bird, 2002). There is contradictory evidence for the role of methylation on allele specific expression in social insects. Methylation is associated with allele specific expression in a number of loci in the ants *Camponotus floridanus* and *Harpegnathos saltator* (Bonasio et al., 2012). Recently, we found evidence for allele specific expression in bumblebee worker reproduction genes (Amarasinghe et al., 2015) and that methylation is important in bumblebee worker reproduction (Amarasinghe, Clayton & Mallon, 2014). However, other work on the honeybee *Apis mellifera* found no link between genes showing allele specific expression and known methylation sites in that species (Kocher et al., 2015).

The presence of allele specific expression does not necessarily mean an epigenetic process is involved. Allele specific expression is known to be caused by a number of genetic as well as epigenetic processes (Palacios et al., 2009). The genetic process usually involves cis effects such as transcription factor binding sites, or less often, untranslated regions which alter RNA stability or microRNA binding (Farh et al., 2005).

The aim of this paper is to investigate allele specific expression and methylation in the bumblebee, *Bombus terrestris*. The recently sequenced genome of the bumblebee, *Bombus terrestris* displays a full complement of genes involved in the methylation system (Sadd et al., 2015). An extreme form of allele specific expression involves monoallelic expression, where one allele is completely silenced. In the canonical mammal and flowering plant systems, this is often associated with monoallelic methylation. In this paper, we examined the link between monoallelic methylation and monoallelic expression in the bumblebee, *Bombus terrestris* using an integrative approached previously used in human epigenetic studies (Harris et al., 2010). Namely, we compare two types of whole methylome libraries and an RNA-seq library from the same individual. In humans, this integrative approach has been independently validated by clonal bisulphite sequencing (Harris et al., 2010). MeDIP-seq is an immunoprecipitation technique that creates libraries enriched for methylated cytosines (Harris et al., 2010). Methyl-sensitive restriction enzymes can create libraries that are enriched for non-methylated cytosines (MRE-seq) (Harris et al., 2010). Genes found in both libraries are predicted to be monoallelically methylated, with the putatively hypermethylated allele being in the MeDIP-seq data and the putatively hypomethylated allele in the MRE-seq data (Harris et al., 2010). Monoallelic expression was identified in these loci from the RNA-seq library. If only one allele was expressed then we knew that these loci were both monoallelically methylated and monoallelically expressed in this bee. We confirmed this monoallelic expression in one locus using qPCR.

We then more generally searched for allele specific expression by analysing twenty nine published RNA-seq libraries from worker bumblebees (Harrison, Hammond & Mallon, 2015; Riddell et al., 2014). We identified heterozygotes in the RNA-seq libraries and measured the expression of each allele. We then identified loci that showed significant expression differences between their two alleles.

## Materials and Methods

### Samples

Data from twenty-nine RNA-seq libraries were used for the allele specific expression analysis (six from Harrison, Hammond & Mallon (2015), and twenty-three from Riddell et al. (2014)). The Riddell bees came from two colonies, one commercially reared bumblebee colony from Koppert Biological Systems UK and one colony from a wild caught queen from the botanic gardens, Leicester. The Harrison bees were from four commercially reared colonies obtained from Agralan Ltd. A Koppert colony worker bee was used for the MeDIP-seq / MRE-seq / RNA-seq experiment. Bees from three different Koppert colonies were used for the qPCR analysis. Samples are outlined in Table 1. Colonies were fed *ad libitum* with pollen (Percie du sert, France) and 50% diluted glucose/fructose mix (Meliose—Roquette, France). Before and during the experiments colonies were kept at 26 °C and 60% humidity in constant red light.

**Table 1:** Bees used in each experiment. K refers to Koppert, A to Agralan and Q to the wild caught Leicester queen.

Experiment	Number	Colony	Tissue
Allele specific expression RNA-seq	1	A1	Whole body
	2	A2	Whole body
	2	A3	Whole body
	1	A4	Whole body
	14	K1	Abdomen
	9	Q1	Abdomen
MeDip/MRE/RNA-seq	1	K2	Whole body
qPCR	2	K3	Head
	1	K4	Head
	1	K5	Head

### Next generation sequencing

#### MeDIP-seq, MRE-seq and RNA-seq

RNA and DNA was extracted from a single five day old whole bee (Colony K2). DNA was extracted using an ethanol precipitation method. Total RNA was extracted using Tri-reagent (Sigma-Aldrich, UK).

Three libraries were prepared from this bee by Eurofins genomics. These were MeDIP-seq and MRE-seq libraries on the DNA sample and one amplified short insert cDNA library with size of 150–400 bp on the RNA sample. Both the MeDIP-seq and MRE-seq library preparations are based on previously published protocols (Harris et al., 2010). MeDIP-seq uses monoclonal antibodies against 5-methylcytosine to enrich for methylated DNA independent of DNA sequence. MRE-seq enriches for unmethylated cytosines by using methylation-sensitive enzymes that cut only restriction sites with unmethylated CpGs. Each library was individually indexed. Sequencing was performed on an Illumina HiSeq^®^2000 instrument (Illumina, Inc., San Diego, CA, USA) by the manufacturer’s protocol. Multiplexed 100 base paired-read runs were carried out yielding 9,390 Mbp for the MeDIP-seq library, 11,597 Mbp for the MRE-seq library and 8,638 Mbp for the RNA-seq library.

#### Previously published RNA-seq

Full details of the RNA-seq protocols used have been published previously (Harrison, Hammond & Mallon, 2015; Riddell et al., 2014). Briefly, for the Riddell bees, total RNA was extracted from twenty three individual homogenised abdomens using Tri-reagent (Sigma-Aldrich, Irvine, UK). TruSeq RNA-seq libraries were made from the 23 samples at NBAF Edinburgh. Multiplexed 50 base single-read runs was performed on an Illumina HiSeq2000 instrument (Illumina, Inc.) by the manufacturer’s protocol. For the Harrison bees, total RNA was extracted from whole bodies using a GenElute Mammalian Total RNA Miniprep kit (Sigma-Aldrich) following the manufacturers’ protocol. The six libraries were sequenced as multiplexed 50 base single-read runs on an Illumina HiSeq 2500 system in rapid mode at the Edinburgh Genomics facility of the University of Edinburgh.

### Monoallelic methylation and expression—bioinformatic analysis

We searched for genes that were monoallelically methylated (present in both MeDip-seq (the putatively hypermethylated allele) and MRE-seq (the putatively hypomethylated allele) libraries), heterozygous (different alleles in the methylation libraries) and monoallelically expressed (only one allele present in the RNA-seq library).

#### Alignment and bam refinement

mRNA reads were aligned to the *Bombus terrestris* genome assembly (AELG00000000) using Tophat (Kim et al., 2013) and converted to bam files with Samtools (Li et al., 2009). Reads were labelled with the AddOrReplaceReadGroups.jar utility in Picard (http://picard.sourceforge.net/). The MRE-seq and MeDIP-seq reads were aligned to the genome using BWA mapper (Li & Durbin, 2009). The resultant sam alignments were soft-clipped with the CleanSam.jar utility in Picard and converted to bam format with Samtools. The Picard utility AddOrReplaceReadGroups.jar was used to label the MRE and MeDIP reads which were then locally re-aligned with GATK (DePristo et al., 2011; McKenna et al., 2010). PCR duplicates for all bams (mRNA, MeDIP and MRE) were marked with the Picard utility Markduplicates.jar.

#### Identifying regions of interest and integrating data

Coverage of each data type was calculated using GATK DepthofCoverage (McKenna et al., 2010). Only regions with a read depth of at least six in each of the libraries (RNA-seq, MeDIP-seq and MRE-seq) was used. Heterozygotes were identified using Samtools mpileup and bcftools on each data set separately (Li & Durbin, 2009) and results were merged with vcf tools (Danecek et al., 2011). Regions of mRNA with overlaps of MeDIP, MRE, and monoallelic snps were identified with custom perl scripts.

### Allele specific expression—bioinformatic analysis

We created a pipeline to search for heterozygous loci that show allele specific expression and identify the associated enriched gene ontology (GO) terms in twenty-nine previously published RNA-seq libraries (Harrison, Hammond & Mallon, 2015; Riddell et al., 2014).

Each RNA library was mapped to the *Bombus terrestris* reference genome (Bter 1.0, accession AELG00000000.1) (Sadd et al., 2015) using the BWA mapper (Li & Durbin, 2009). The combat method in the R package SVA (version 3.20.0) was used to remove any batch effects and control for original differences in coverage (Leek et al., 2012; Johnson, Li & Rabinovic, 2007). The success of this control was confirmed by the R package edgeR (version 3.14.0) (McCarthy, Chen & Smyth, 2012; Robinson, McCarthy & Smyth, 2010).

Bcftools (version 0.1.19-44428cd), bedtools (version 2.17.0), and SAMtools (version 0.1.19-44428cd) were used to prepare the RNA libraries and call the SNPs, before the SNPs were filtered based on mapping quality score (Quinlan & Hall, 2010; Li & Durbin, 2009). Only SNPs with a mapping quality score of *p* < 0.05 and a read depth of ≥6 were included in the analyses.

The R package, QuASAR implements a statistical method for: (1) genotyping from next-generation sequencing reads (according to the Hardy–Weinberg equilibrium), and (2) conducting inference on allele specific expression at heterozygous sites (Harvey et al., 2015). One problem with genotyping heterozygotes is being able to identical true homozygotes that appear heterozygote due to base-calling errors. QuASAR removes snps with extreme differential allele expression from the analyses, thus controlling for any base-calling errors. Despite this inherent conservatism, in benchmark tests, QuaSAR can accurately genotype loci with lower error rates than other methods commonly used for genotyping DNA-seq data (Harvey et al., 2015). The allele specific expression inference step takes into consideration the uncertainty in the genotype calls, base-call errors in sequencing, and allelic over-dispersion. QuASAR is a powerful tool for detecting allele specific expression if, as during most RNA-seq experiments, genotypes are not available (Harvey et al., 2015).

Sequence regions (the snp position +∕ − 2,900 bp), encompassing the loci identified as showing ASE in at least three of the thirty libraries, were compared to *Drosophila melanogaster* proteins (non-redundant (nr) database) with Blastx (Altschul et al., 1997). The blast results were annotated using Blast2Go (Gotz et al., 2008). We carried out an enrichment analysis (Fisher exact test) using a custom R script (https://dx.doi.org/10.6084/m9.figshare.3201355.v1) on this list of GO terms. This identified GO terms that are overrepresented (*p* < 0.05) relative to the entire bumblebee transcriptome (https://dx.doi.org/10.6084/m9.figshare.3458828.v1). We then used REVIGO to summarize and visualise these terms (Supek et al., 2011). REVIGO summarizes lists of GO terms using a clustering algorithm based on semantic similarity measures. To identify which bumblebee genes the snps were located in, the snp position +∕ − 25 bp was compared against the *Bombus terrestris* genome (Sadd et al., 2015) using Blastn.

### Candidate gene allele specific qPCR

DNA was extracted from four bees from three Koppert colonies using the Qiagen DNA Micro kit according to manufacturer’s instructions. RNA was extracted from samples of the heads of the same worker bees with the QIAGEN RNeasy Mini Kit according to manufacturer’s instructions. cDNA was synthesized from a 8 µl sample of RNA using the Tetro cDNA synthesis Kit (Bioline, London, UK) as per manufacturer’s instructions.

We amplified numerous fragments of the 19 candidate genes. Sanger sequencing results were analyzed using the heterozygote analysis module in Geneious version 7.3.0 to identify heterozygotic nucleotide positions. It was difficult to identify snps in exonic regions of the 19 loci, which could be amplified with primers of suitable efficiency. We managed to identify a suitable region in *toll-like receptor Tollo* (AELG01000623.1 exonic region 1838–2420).

The locus was run for three different reactions; T allele, G allele and reference. Reference primers were designed according to Gineikiene, Stoskus & Griskevicius (2009). A common reverse primer (CTGGTTCCCGTCCAATCTAA) was used for all three reactions. A reference forward primer (CGTGTCCAGAATCGACAATG) was designed to the same target heterozygote sequence, upstream of the heterozygote nucleotide position. The reference primers measure the total expression of the gene, whereas the allele specific primers (T allele: CCAGAATCGACAATGACTCGT, G allele: CAGAATCGACAATGACTCGG) measure the amount of expression due to the allele. Thus the ratio between the allele specific expression and reference locus expression would be the relative expression due to the allele.

Three replicate samples were run for each reaction. All reactions were prepared by the Corbett robotics machine, in 96 well qPCR plates (Thermo Scientific, Loughborough, UK). The qPCR reaction mix (20 µl) was composed of 1 µl of diluted cDNA (50 ng/ µl), 1 µl of forward and reverse primer (5 µM/ µl each), 10 µl 2X SYBR Green JumpStart Taq ReadyMix (Sigma Aldrich, Irvine, UK) and 7 µl ddH_2_0. Samples were run in a PTC-200 MJ thermocycler. The qPCR profile was; 4 min at 95 °C denaturation followed by 40 cycles of 30 s at 95 °C, 30 s at 59 °C and 30 s at 72 °C and a final extension of 5 min at 72 °C.

Forward primers are different, both in their terminal base (to match the snp) and in their length. It is entirely possible that they may amplify more or less efficiently even if there was no difference in amount of template (Pfaffl, 2001). To test for this we repeated all qPCRs with genomic DNA (1 µl of diluted DNA (20 ng/ µl) from the same bees as the template. We would expect equal amounts of each allele in the genomic DNA. We also measured efficiency of each reaction as per Liu & Saint (2002).

Median *C*_*t*_ was calculated for each set of three technical replicates. A measure of relative expression (ratio) was calculated for each allele in each worker bee as follows:

(1) \begin{eqnarray*}{ratio}_{allele}= \frac{{E}_{allele}^{-C{t}_{allele}}}{{E}_{reference}^{-C{t}_{reference}}} \end{eqnarray*}

*E* is the median efficiency of each primer set (Liu & Saint, 2002; Pfaffl, 2001). All statistical analysis was carried out using R (3.3.1) (R core Team, 2016).

## Results

### Discovery of monoallelically methylated and expressed genes

**Figure 1:**
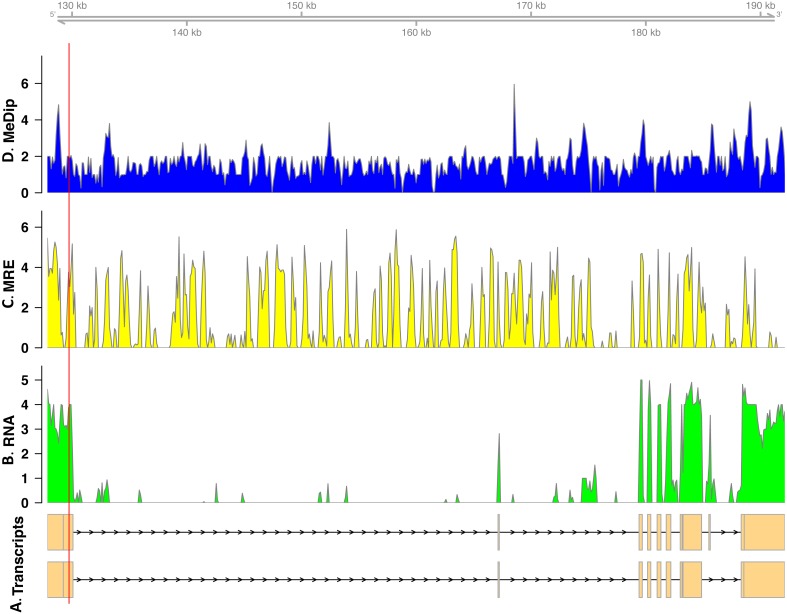
Coverage of the three libraries for *ras GTPase-activating protein nGAP-like* (LOC100652225). The transcript models come from GCF_000214255.1_Bter_1.0. The *y*-axis in the coverage plots is log (1 + coverage). The red vertical line represents the heterozygote position. The MeDip allele was expressed in this locus, see Table 2.

**Figure 2:**
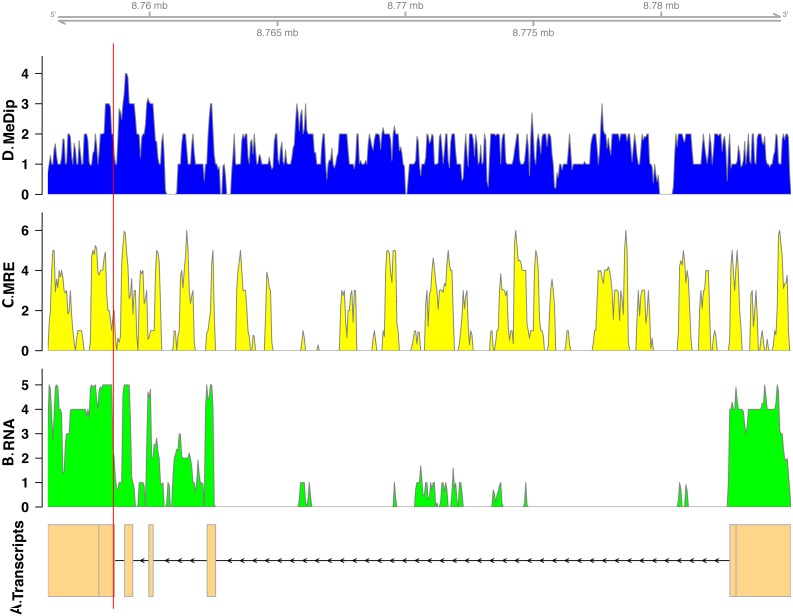
Coverage of the three libraries for *bicaudal D-related protein homolog* ( LOC100650109). The transcript model come from GCF_000214255.1_Bter_1.0. The *y*-axis in the coverage plots is log (1 + coverage). The red vertical line represents the heterozygote position. The MeDip allele was expressed in this locus, see Table 2.

In total, we found nineteen genes that were both monoallelically methylated (present in both Me-DIP and MRE-seq libraries) and monoallelically expressed (only one allele present in the RNA-seq library). Figures 1 and 2 show the coverage of the three libraries for two examples of these genes (*ras GTPase-activating protein nGAP-like* and *bicaudal-D*). Of the nineteen genes, fourteen had the hypermethylated (MeDIP) allele expressed, while five had the hypomethylated (MRE-seq) allele expressed (see Tables S1). The nineteen genes were compared to the nr/nt database using Blastn. Six returned noninformative hits (Table 2).

**Table 2:** The thirteen of the nineteen monoallelically methylated and expressed genes that returned informative blast hits.

Gene	Accession	Expressed allele	Function
*yippee-like 1*	LOC100642754	MeDIP	Yippee is an intracellular protein with a zinc-finger like domain. DNA methylation of a CpG island near the *yippie-like 3* promoter in humans represents a possible epigenetic mechanism leading to decreased gene expression in tumours (Kelley et al., 2010).
*toll-like receptor Tollo*	LOC100644648	MeDIP	Tollo regulates antimicrobial response in the insect respiratory epithelium (Akhouayri et al., 2011).
*zinc finger protein Elbow*	LOC100650465	MeDIP	The elbow (elB) gene is involved in the formation of the insect tracheal system (Dorfman et al., 2002).
*heterogeneous nuclear ribonucleoprotein A3*	LOC100651168	MeDIP	Heterogeneous nuclear ribonucleoproteins associated with precursors of functional, protein coding mRNAs (Dreyfuss et al., 1993).
*calmodulin-lysine N-methyltransferase-like*	LOC100749522	MRE	Calmodulin-lysine N-methyltransferase catalyses the trimethylation of a lysine residue of calmodulin. Calmodulin is a ubiquitous, calcium-dependent, eukaryotic signalling protein with a large number of interactors. The methylation state of calmodulin causes phenotypic changes in growth and developmental processes (Magnani et al., 2010).
*Na/K/Ca exchanger CG1090*	LOC107998466	MRE	CG1090 functions in the maintenance of calcium homeostasis.
*Shaker*	LOC100648438	MeDIP	Shaker is involved in the operation of potassium ion channel. *Shaker* expression was upregulated in sterile versus reproductive honeybee workers (Cardoen et al., 2011).
*Centrosomal and chromosomal factor-like*	LOC105665737	MeDIP	Essential protein required for proper condensation of mitotic chromosomes and progression through mitosis. Expressed during oogenesis in *Drosophila* (Kodjabachian et al., 1998).
*excitatory amino acid transporter 1*	LOC100744217	MRE	Excitatory amino acid transporters are neurotransmitter transporters. *Excitatory amino acid transporter 3* expression was upregulated in sterile honeybee workers (Cardoen et al., 2011). *Excitatory amino acid transporter 1* expression differences were also associated with worker - queen differentiation in the paper wasp *Polistes metricus* (Toth et al., 2014).
*aminopeptidase M1-like*	LOC105666993	MeDIP	M1 aminopeptidases are zinc-dependent enzymes that catalyze the removal of amino acids from the N terminus of polypeptides (Drinkwater et al., 2017).
*ras GTPase-activating protein nGAP-like*	LOC100652225	MeDIP	*Ras GTPase-activating protein 1* was found to be upregulated in reproductive honeybee workers (Cardoen et al., 2011). It is involved in oocyte meiosis.
*neuromedin-B receptor-like*	LOC100745453	MeDIP	In humans, this G protein-coupled receptor binds neuromedin B, a peptide that stimulates mitosis in gastrointestinal epithelial tissue.
*bicaudal D-related protein homolog*	LOC100650109	MeDIP	Bicaudal is involved in embryonic pattern formation in *Drosophila* (Markesich et al., 2000). It is thought to be involved in the differentiation between soldiers and workers in the termite *Reticulitermes flavipes* (Scharf et al., 2003). *Bicaudal protein D* has been shown to be methylated more in eggs than sperm in honeybees (Drewell et al., 2014).

### Confirmation of monoallelic expression

Monoallelic expression was confirmed in one of these nineteen (*toll-like receptor Tollo* (LOC100644648)) by allele specific qPCR (Amarasinghe et al., 2015). The allele with a guanine at the snp position had a mean expression of 6.04 ± 8.28 (standard deviation) in four bees from three different colonies. The thymine allele was not expressed at all in these bees. This was not due to the efficiency of the primers as the DNA controls of both alleles showed similar amplification (G mean =422.70 ± 507.36, T mean =1575.17 ± 503.02). In the three other loci tested (*Ras GTPase-activating protein 1*, LOC107964816, *Elbow*) we found apparent monoallelic expression, but could not dismiss primer efficiency as the cause.

We then looked at these nineteen genes in twenty-nine previously published RNA-seq libraries. Fifteen of these nineteen genes expressed a single allele in all twenty nine RNA-seq libraries, see Tables S2. The remaining four genes were inconsistent; they showed expression of one allele in some *B. terrestris* workers, and expression of two alleles in other workers.

### Removing batch effects

The twenty nine RNA-seq libraries do not come from the same experiment (Table 1). This gives rise to the possibility of batch effects, sources of variation due to samples not being from the same source or not being run together. We must remove these before any other analysis.

The mean GC content of the 29 libraries was 42.34%, with individual libraries having a similar GC content ranging from 40–46%. GC content differed with run (Nested ANOVA: *F* = 20.302, *df* = 1, *p* < 0.001), but not by colony (Nested ANOVA: *F* = 1.763, *df* = 4, *p* = 0.171). The mean coverage of the 29 libraries was 13.29, with mean library coverage ranging from 9.84 to 17.61. Run had an effect on coverage (Nested ANOVA: *F* = 7.554, *df* = 1, *p* = 0.011), as did colony (Nested ANOVA: *F* = 6.962, *df* = 4, *p* < 0.001).

**Figure 3:**
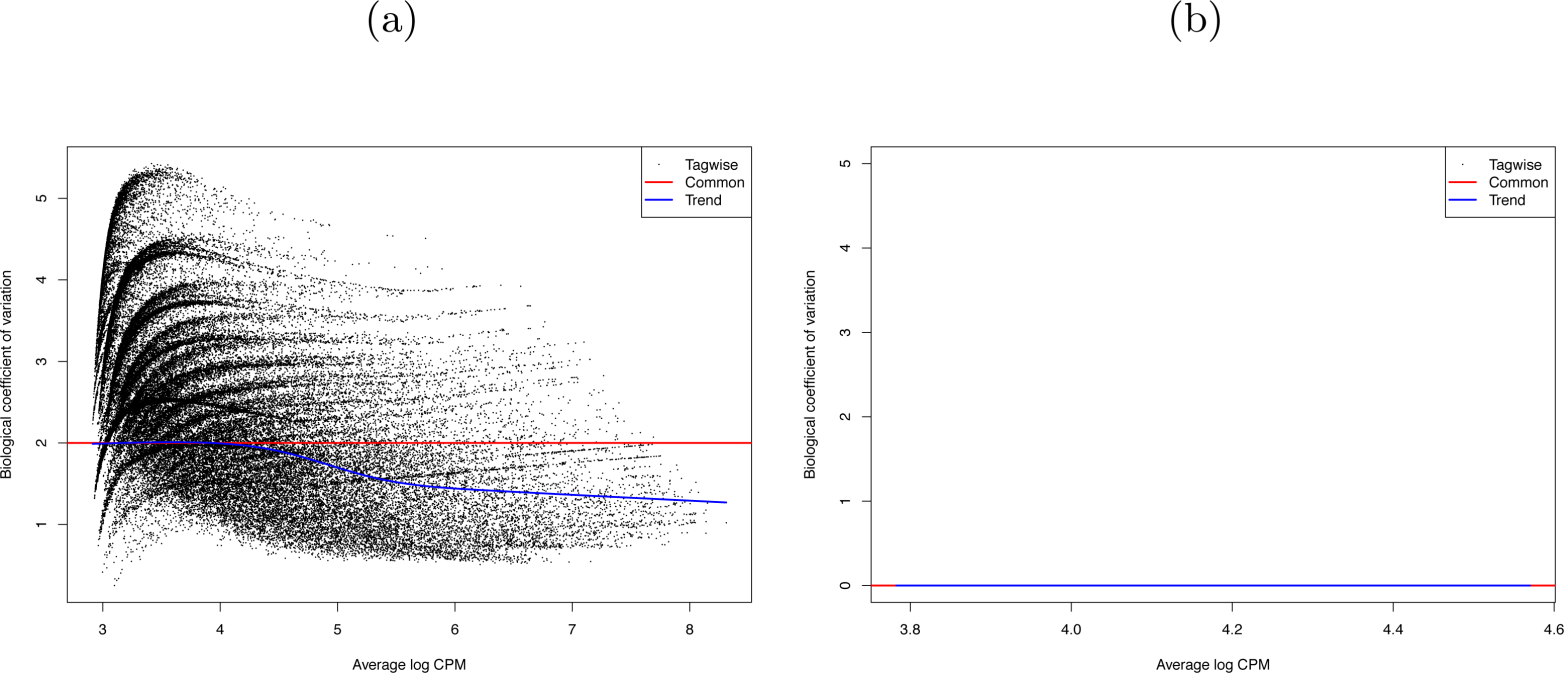
Biological coefficient of variation (BCV) of (A) raw data, and (B) SVA-adjusted data for the 29 RNA-seq *Bombus terrestris* libraries. The black dots represent the BCV if it were calculated individually for each gene (tagwise). The blue line is the trend of this data. The red line represents the BCV of the samples if a common dispersion value, over all genes, were used. In (B) tagwise values are exactly the same as common values so no black dots are visible.

Therefore, the combat method in the R package SVA (version 3.20.0) was used to remove any batch effects and control for original differences in coverage (Leek et al., 2012; Johnson, Li & Rabinovic, 2007). The success of this control was confirmed by the R package edgeR (version 3.14.0) (McCarthy, Chen & Smyth, 2012; Robinson, McCarthy & Smyth, 2010). The SVA adjustment reduced the edgeR dispersion value from 3.9994 (BCV  = 2) to 0 (BCV  = 0.0003) (see Fig. 3). That is, we successfully removed the batch effects due to the separate runs.

### Allele specific expression—RNA-seq

**Figure 4:**
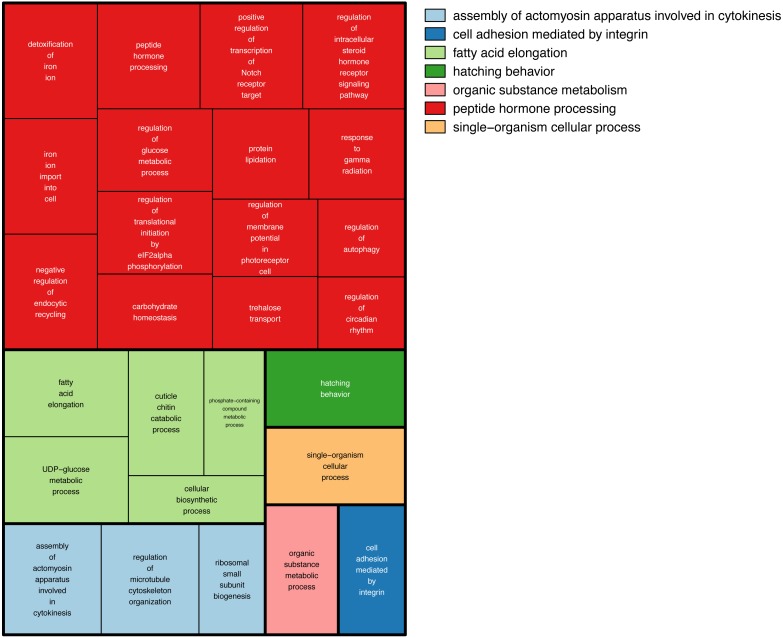
GO terms associated with allele specific expression. A summary of the enriched GO terms (*p* < 0.05, based on Blast2Go annotation) found for genes displaying allele specific expression. This figure was produced using Revigo (Supek et al., 2011). Each rectangle represents a single cluster of closely related GO terms. These rectangles are joined into different coloured ‘superclusters’ of loosely related terms. The area of the rectangles represents the *p*-value associated with that cluster’s enrichment.

We then searched more generally for allele specific expression in the twenty-nine RNA-seq libraries. A total of 555 loci showed allele-specific expression in ≥3 of the 29 RNA-seq libraries (Tables S3). Comparing these loci against the *Bombus terrestris* genome using Blastn returned 211 hits. To search for gene ontology terms, we compared them against *Drosophila melanogaster* proteins, using Blastx, which returned 329 hits. We tested for enriched gene ontology (GO) terms against their background value in the bumblebee transcriptome. One hundred and fifty-one Gene Ontology(GO) terms were enriched in the 555 regions showing allele specific expression (Fisher’s exact test *p* > 0.05), however none were significant at the more stringent FDR > 0.05. Figure 4 shows the large number of biological functions associated with these 555 genes.

## Discussion

An important caveat about the integrative analysis of monoallelic methylation and expression carried out here is that all three libraries were from a single bee. It is certain that there is variation in methylation and allele specific expression between bees just as there is in other species (Pignatta et al., 2014). We attempted to confirm this monoallelic expression in other bees using RNA-seq and qPCR but with limited success. This analysis is only a first step in understanding the link between monoallelic methylation and expression.

Of the nineteen genes displaying monoallelic methylation and monoallelic expression, fourteen had the hypermethylated (MeDIP) allele expressed, while five had the hypomethylated (MRE-seq) allele expressed (see Tables S1). In ant genes with allele specific methylation, the hypermethylated allele showed more expression than the hypomethylated allele (Bonasio et al., 2012). This fits with genome wide analysis that shows exonic methylation in insects associated with increased gene expression (Glastad, Hunt & Goodisman, 2014; Yan et al., 2015). Our fourteen genes with the hypermethylated allele expressed agree with this pattern. But how to explain the five genes where the hypomethylated allele was expressed? Firstly, the role of methylation in insect gene expression is not clear cut, with the relationship between exonic methylation and expression often disappearing at the gene level (Yan et al., 2015). For example, *EGFR* expression is lower in ant workers that exhibit higher DNA methylation of *EGFR* (Alvarado et al., 2015). Secondly, even in the canonical mammalian methylation system, the “wrong” allele has been shown to be expressed occasionally due to lineage specific effects (Dean et al., 1998; Pardo-Manuel de Villena, de la Casa-Esperón & Sapienza, 2000; Onyango et al., 2002; Sapienza, 2002; Zhang et al., 1993).

We analysed RNA-seq libraries from different published sources. This lead to two confounding problems. The first is that as the samples were run at different times, using different machines this could lead to a batch effect. We were able to successfully remove this. The second, that the libraries were made from abdomens in some cases and whole bodies in others, is still a confounding effect. Allele specific expression is known to vary between tissues (Chamberlain et al., 2015). Any variation in which allele is expressed could be due to these tissue effects.

We looked at the expression of the nineteen genes in all twenty-nine RNA-seq libraries. If they are monoallelically expressed in these bees, we would find only one allele in a given RNA-seq library. Fifteen of these nineteen genes were confirmed to show a single allele in all twenty-nine RNA-seq libraries. We would also find only one allele if that bee was homozygous. We cannot rule out that these fifteen genes just happen to be homozygous in all twenty-nine bees from five different colonies from multiple sources.

The remaining four genes showed inconsistent expression with one allele being expressed in some *B. terrestris* workers, and expression of two alleles in other workers. Natural intraspecific variation in allele specific expression has been found in other species (Pignatta et al., 2014). The tissue variation mentioned above is also a possibility. Another explanation is that these loci are not epigenetically controlled but rather their allele specific expression is derived from genetic effects (Remnant et al., 2016).

There are three main genetic, as opposed to epigenetic, affectors of allele specific expression (Edsgard et al., 2016). Allele specific expression can be caused by differences in the alleles’ sequence within the translated part resulting in a modified protein. A change at the alleles’ cis regulatory sites, could cause differential binding of transcription factors. Transcript processing can be affected by a change in the alleles’ sequence a splice site or untranslated region. This large number of possible causes of allele specific expression could explain why we see so many functions associated with the 555 genes showing allele specific expression (Fig. 4).

But it is not just allele specific expression that may have genetic as well as epigenetic effects. It has been shown in humans that some allele specific methylation is determined by DNA sequence in cis and therefore shows Mendelian inheritance patterns (Meaburn, Schalkwyk & Mill, 2010). An extreme example of genetically controlled allele specific methylation is found in *Nasonia* wasps, where there is no evidence for methylation driven allele specific expression but inheritable cis-mediated allele specific methylation has been found (Wang, Werren & Clark, 2016). This cis-mediated methylation has recently been suggested as being important in social insect biology (Remnant et al., 2016; Wedd, Kucharski & Maleszka, 2016).

We have found that allele specific expression is widespread in the bumblebee. We have also found that the extreme version of allele specific expression, monoalleic expression is associated with monoallelic methylation. Genomic imprinting in mammals usually involves monoallelic methylation and expression. Although tempting to associate our results with genomic imprinting, this current work is unable to identify genomic imprinting. In any case, caution should be applied due to the lack of understanding of the functional role of methylation in gene expression in insects and in the, as yet unquantified, role of genetic cis effects in insect allele specific methylation and expression.

##  Supplemental Information

10.7717/peerj.3798/supp-1 Table S1 Nineteen genes showing both monoallelic methylation and monoallelic expression Blast results and genomic coordinates of the reads from the RNA-seq, MRE-seq and MeDip-seq libraries.

10.7717/peerj.3798/supp-2 Table S2 Confirmation of single allele expression of nineteen monoallelically expressed genes in twenty-nine previously published transcriptomes For each of the 19 contigs, the previously published RNA-seq libraries with associated read counts are included.

10.7717/peerj.3798/supp-3 Table S3 555 genes showing allele specific expression in at least three of the 29 previously published RNA-seq libraries This table details the blast results from both the bumblebee and drosophila genomes and the GO terms associated with the drosophila hits.

## Additional Information and Declarations

### Data Availability

The following information was supplied regarding data availability: All sequence data for this study are archived under NCBI BioProject numbers PRJEB9366 and PRJNA391408. GO-analysis results and lists of differentially expressed transcripts are available as Supplemental Information.

## References

[ref-1] Akhouayri I, Turc C, Royet J, Charroux B (2011). Toll-8/Tollo negatively regulates antimicrobial response in the Drosophila respiratory epithelium. PLOS Pathogens.

[ref-2] Altschul SF, Madden TL, Schaffer AA, Zhang JH, Zhang Z, Miller W, Lipman DJ (1997). Gapped BLAST and PSI-BLAST: a new generation of protein database search programs. Nucleic Acids Research.

[ref-3] Alvarado S, Rajakumar R, Abouheif E, Szyf M (2015). Epigenetic variation in the Egfr gene generates quantitative variation in a complex trait in ants. Nature Communications.

[ref-4] Amarasinghe H, Toghill B, Nathanael D, Mallon EB (2015). Allele specific expression in worker reproduction genes in the bumblebee Bombus terrestris. PeerJ.

[ref-5] Amarasinghe HE, Clayton CI, Mallon EB (2014). Methylation and worker reproduction in the bumble-bee (Bombus terrestris). Proceedings of the Royal Society B: Biological Sciences.

[ref-6] Bird A (2002). DNA methylation patterns and epigenetic memory. Genes & Development.

[ref-7] Bonasio R, Li Q, Lian J, Mutti NS, Jin L, Zhao H, Zhang P, Wen P, Xiang H, Ding Y, Jin Z, Shen SS, Wang Z, Wang W, Wang J, Berger SL, Liebig J, Zhang G, Reinberg D (2012). Genome-wide and caste-specific DNA methylomes of the ants camponotus floridanus and harpegnathos saltator. Current Biology.

[ref-8] Cardoen D, Wenseleers T, Ernst UR, Danneels EL, Laget D, DE Graaf DC, Schoofs L, Verleyen P (2011). Genome-wide analysis of alternative reproductive phenotypes in honeybee workers. Molecular Ecology.

[ref-9] Chamberlain AJ, Vander Jagt CJ, Hayes BJ, Khansefid M, Marett LC, Millen CA, Nguyen TTT, Goddard ME (2015). Extensive variation between tissues in allele specific expression in an outbred mammal. BMC Genomics.

[ref-10] Chittka A, Wurm Y, Chittka L (2012). Epigenetics: the making of ant castes. Current Biology.

[ref-11] Danecek P, Auton A, Abecasis G, Albers CA, Banks E, DePristo MA, Handsaker RE, Lunter G, Marth GT, Sherry ST, McVean G, Durbin R (2011). The variant call format and VCFtools. Bioinformatics.

[ref-12] Dean W, Bowden L, Aitchison A, Klose J, Moore T, Meneses JJ, Reik W, Feil R (1998). Altered imprinted gene methylation and expression in completely ES cell-derived mouse fetuses: association with aberrant phenotypes. Development.

[ref-13] DePristo MA, Banks E, Poplin R, Garimella KV, Maguire JR, Hartl C, Philippakis AA, Del Angel G, Rivas MA, Hanna M, McKenna A, Fennell TJ, Kernytsky AM, Sivachenko AY, Cibulskis K, Gabriel SB, Altshuler D, Daly MJ (2011). A framework for variation discovery and genotyping using next-generation DNA sequencing data. Nature Genetics.

[ref-14] Dorfman R, Glazer L, Weihe U, Wernet MF, Shilo B-Z (2002). Elbow and Noc define a family of zinc finger proteins controlling morphogenesis of specific tracheal branches. Development.

[ref-15] Drewell RA, Bush EC, Remnant EJ, Wong GT, Beeler SM, Stringham JL, Lim J, Oldroyd BP (2014). The dynamic DNA methylation cycle from egg to sperm in the honey bee Apis mellifera. Development.

[ref-16] Dreyfuss G, Matunis MJ, Piñol-Roma S, Burd CG (1993). hnRNP proteins and the biogenesis of mRNA. Annual Review of Biochemistry.

[ref-17] Drinkwater N, Lee J, Yang W, Malcolm TR, McGowan S (2017). M1 aminopeptidases as drug targets: broad applications or therapeutic niche?. The FEBS Journal.

[ref-18] Edsgard D, Iglesias MJ, Reilly S-J, Hamsten A, Tornvall P, Odeberg J, Emanuelsson O (2016). GeneiASE: detection of condition-dependent and static allele-specific expression from RNA-seq data without haplotype information. Scientific Reports.

[ref-19] Farh KK-H, Grimson A, Jan C, Lewis BP, Johnston WK, Lim LP, Burge CB, Bartel DP (2005). The widespread impact of mammalian MicroRNAs on mRNA repression and evolution. Science.

[ref-20] Galbraith DA, Kocher SD, Glenn T, Albert I, Hunt GJ, Strassmann JE, Queller DC, Grozinger CM (2016). Testing the kinship theory of intragenomic conflict in honey bees (Apis mellifera). Proceedings of the National Academy of Sciences of the United States of America.

[ref-21] Gineikiene E, Stoskus M, Griskevicius L (2009). Single nucleotide polymorphism-based system improves the applicability of quantitative PCR for chimerism monitoring. The Journal of Molecular Diagnostics.

[ref-22] Glastad KM, Hunt BG, Goodisman MA (2014). Evolutionary insights into DNA methylation in insects. Current Opinion in Insect Science.

[ref-23] Glastad KM, Hunt BG, Yi SV, Goodisman MAD (2011). DNA methylation in insects: on the brink of the epigenomic era. Insect Molecular Biology.

[ref-24] Goldberg A, Allis C, Bernstein E (2007). Epigenetics: a landscape takes shape. Cell.

[ref-25] Gotz S, García-Gómez JM, Terol J, Williams TD, Nagaraj SH, Nueda MJ, Robles M, Talón M, Dopazo J, Conesa A (2008). High-throughput functional annotation and data mining with the Blast2GO suite. Nucleic Acids Research.

[ref-26] Harris RA, Wang T, Coarfa C, Nagarajan RP, Hong C, Downey SL, Johnson BE, Fouse SD, Delaney A, Zhao Y, Olshen A, Ballinger T, Zhou X, Forsberg KJ, Gu J, Echipare L, O’Geen H, Lister R, Pelizzola M, Xi Y, Epstein CB, Bernstein BE, Hawkins RD, Ren B, Chung W-Y, Gu H, Bock C, Gnirke A, Zhang MQ, Haussler D, Ecker JR, Li W, Farnham PJ, Waterland RA, Meissner A, Marra MA, Hirst M, Milosavljevic A, Costello JF (2010). Comparison of sequencing-based methods to profile DNA methylation and identification of monoallelic epigenetic modifications. Nature Biotechnology.

[ref-27] Harrison MC, Hammond RL, Mallon EB (2015). Reproductive workers show queen-like gene expression in an intermediately eusocial insect, the buff-tailed bumble bee Bombus terrestris. Molecular Ecology.

[ref-28] Harvey CT, Moyerbrailean GA, Davis GO, Wen X, Luca F, Pique-Regi R (2015). QuASAR: quantitative allele-specific analysis of reads. Bioinformatics.

[ref-29] Johnson WE, Li C, Rabinovic A (2007). Adjusting batch effects in microarray expression data using empirical Bayes methods. Biostatistics.

[ref-30] Kelley K, Miller KR, Todd A, Kelley A, Tuttle R, Berberich SJ (2010). YPEL3, a p53-regulated gene that induces cellular senescence. Cancer Research.

[ref-31] Kim D, Pertea G, Trapnell C, Pimentel H, Kelley R, Salzberg SL (2013). TopHat2: accurate alignment of transcriptomes in the presence of insertions, deletions and gene fusions. Genome Biology.

[ref-32] Kocher SD, Tsuruda JM, Gibson JD, Emore CM, Arechavaleta-Velasco ME, Queller DC, Strassmann JE, Grozinger CM, Gribskov MR, San Miguel P, Westerman R, Hunt GJ (2015). A search for parent-of-origin effects on honey bee gene expression. G3.

[ref-33] Kodjabachian L, Delaage M, Maurel C, Miassod R, Jacq B, Rosset R (1998). Mutations in ccf, a novel Drosophila gene encoding a chromosomal factor, affect progression through mitosis and interact with Pc-G mutations. The EMBO Journal.

[ref-34] Leek JT, Johnson WE, Parker HS, Jaffe AE, Storey JD (2012). The sva package for removing batch effects and other unwanted variation in high-throughput experiments. Bioinformatics.

[ref-35] Li H, Durbin R (2009). Fast and accurate short read alignment with Burrows-Wheeler transform. Bioinformatics.

[ref-36] Li H, Handsaker B, Wysoker A, Fennell T, Ruan J, Homer N, Marth G, Abecasis G, Durbin R, 1000 Genome Project Data Processing Subgroup (2009). The sequence alignment/map format and SAM tools. Bioinformatics.

[ref-37] Liu W, Saint DA (2002). A new quantitative method of real time reverse transcription polymerase chain reaction assay based on simulation of polymerase chain reaction kinetics. Analytical Biochemistry.

[ref-38] Magnani R, Dirk LMA, Trievel RC, Houtz RL (2010). Calmodulin methyltransferase is an evolutionarily conserved enzyme that trimethylates Lys-115 in calmodulin. Nature Communications.

[ref-39] Markesich DC, Gajewski KM, Nazimiec ME, Beckingham K (2000). bicaudal encodes the Drosophila beta NAC homolog, a component of the ribosomal translational machinery*. Development.

[ref-40] McCarthy DJ, Chen Y, Smyth GK (2012). Differential expression analysis of multifactor RNA-Seq experiments with respect to biological variation. Nucleic Acids Research.

[ref-41] McKenna A, Hanna M, Banks E, Sivachenko A, Cibulskis K, Kernytsky A, Garimella K, Altshuler D, Gabriel S, Daly M, DePristo MA (2010). The genome analysis toolkit: a mapReduce framework for analyzing next-generation DNA sequencing data. Genome Research.

[ref-42] Meaburn EL, Schalkwyk LC, Mill J (2010). Allele-specific methylation in the human genome: implications for genetic studies of complex disease. Epigenetics.

[ref-43] Onyango P, Jiang S, Uejima H, Shamblott MJ, Gearhart JD, Cui H, Feinberg AP (2002). Monoallelic expression and methylation of imprinted genes in human and mouse embryonic germ cell lineages. Proceedings of the National Academy of Sciences of the United States of America.

[ref-44] Palacios R, Gazave E, Goñi J, Piedrafita G, Fernando O, Navarro A, Villoslada P (2009). Allele-specific gene expression is widespread across the genome and biological processes. PLOS ONE.

[ref-45] Pardo-Manuel de Villena F, De la Casa-Esperón E, Sapienza C (2000). Natural selection and the function of genome imprinting: beyond the silenced minority. Trends in Genetics.

[ref-46] Pfaffl MW (2001). A new mathematical model for relative quantification in real-time RT–PCR. Nucleic Acids Research.

[ref-47] Pignatta D, Erdmann RM, Scheer E, Picard CL, Bell GW, Gehring M (2014). Natural epigenetic polymorphisms lead to intraspecific variation in Arabidopsis gene imprinting. ELife.

[ref-48] Queller DC (2003). Theory of genomic imprinting conflict in social insects. Bmc Evolutionary Biology.

[ref-49] Quinlan AR, Hall IM (2010). BEDTools: a flexible suite of utilities for comparing genomic features. Bioinformatics.

[ref-50] R core Team (2016). https://www.R-project.org/.

[ref-51] Reik W, Walter J (2001). Genomic imprinting: parental influence on the genome. Nature Reviews Genetics.

[ref-52] Remnant EJ, Ashe A, Young PE, Buchmann G, Beekman M, Allsopp MH, Suter CM, Drewell RA, Oldroyd BP (2016). Parent-of-origin effects on genome-wide DNA methylation in the Cape honey bee (Apis mellifera capensis) may be confounded by allele-specific methylation. BMC Genomics.

[ref-53] Riddell CE, Garces JDL, Adams S, Barribeau SM, Twell D, Mallon EB (2014). Differential gene expression and alternative splicing in insect immune specificity. BMC Genomics.

[ref-54] Robinson MD, McCarthy DJ, Smyth GK (2010). edgeR: a bioconductor package for differential expression analysis of digital gene expression data. Bioinformatics.

[ref-55] Sadd BM, Barribeau SM, Bloch G, Graaf DCD, Dearden P, Elsik CG, Gadau J, Grimmelikhuijzen CJ, Hasselmann M, Lozier JD, Robertson HM, Smagghe G, Stolle E, Vaerenbergh MV, Waterhouse RM, Bornberg-Bauer E, Klasberg S, Bennett AK, Câmara F, Guigó R, Hoff K, Mariotti M, Munoz-Torres M, Murphy T, Santesmasses D, Amdam GV, Beckers M, Beye M, Biewer M, Bitondi MM, Blaxter ML, Bourke AF, Brown MJ, Buechel SD, Cameron R, Cappelle K, Carolan JC, Christiaens O, Ciborowski KL, Clarke DF, Colgan TJ, Collins DH, Cridge AG, Dalmay T, Dreier S, Plessis LD, Duncan E, Erler S, Evans J, Falcon T, Flores K, Freitas FC, Fuchikawa T, Gempe T, Hartfelder K, Hauser F, Helbing S, Humann FC, Irvine F, Jermiin LS, Johnson CE, Johnson RM, Jones AK, Kadowaki T, Kidner JH, Koch V, Köhler A, Kraus FB, Lattorff HM, Leask M, Lockett GA, Mallon EB, Antonio DSM, Marxer M, Meeus I, Moritz RF, Nair A, Näpflin K, Nissen I, Niu J, Nunes FM, Oakeshott JG, Osborne A, Otte M, Pinheiro DG, Rossié N, Rueppell O, Santos CG, Schmid-Hempel R, Schmitt BD, Schulte C, Simões ZL, Soares MP, Swevers L, Winnebeck EC, Wolschin F, Yu N, Zdobnov EM, Aqrawi PK, Blankenburg KP, Coyle M, Francisco L, Hernandez AG, Holder M, Hudson ME, Jackson L, Jayaseelan J, Joshi V, Kovar C, Lee SL, Mata R, Mathew T, Newsham IF, Ngo R, Okwuonu G, Pham C, Pu L-L, Saada N, Santibanez J, Simmons D, Thornton R, Venkat A, Walden KK, Wu Y-Q, Debyser G, Devreese B, Asher C, Blommaert J, Chipman AD, Chittka L, Fouks B, Liu J, O’Neill MP, Sumner S, Puiu D, Qu J, Salzberg SL, Scherer SE, Muzny DM, Richards S, Robinson GE, Gibbs RA, Schmid-Hempel P, Worley KC (2015). The genomes of two key bumblebee species with primitive eusocial organization. Genome Biology.

[ref-56] Sapienza C (2002). Imprinted gene expression, transplantation medicine, and the other human embryonic stem cell. Proceedings of the National Academy of Sciences of the United States of America.

[ref-57] Scharf ME, Wu-Scharf D, Pittendrigh BR, Bennett GW (2003). Caste and development-associated gene expression in a lower termite. Genome Biology.

[ref-58] Supek F, Bošnjak M, Škunca N, Šmuc T (2011). REVIGO summarizes and visualizes long lists of gene ontology terms. PLOS ONE.

[ref-59] Toth AL, Tooker JF, Radhakrishnan S, Minard R, Henshaw MT, Grozinger CM (2014). Shared genes related to aggression, rather than chemical communication, are associated with reproductive dominance in paper wasps (Polistes metricus). BMC Genomics.

[ref-60] Wang X, Werren JH, Clark AG (2016). Allele-specific transcriptome and methylome analysis reveals stable inheritance and Cis-regulation of DNA methylation in nasonia. PLOS Biology.

[ref-61] Wedd L, Kucharski R, Maleszka R (2016). Differentially methylated obligatory epialleles modulate context-dependent LAM gene expression in the honeybee Apis mellifera. Epigenetics.

[ref-62] Weiner SA, Toth AL (2012). Epigenetics in social insects: a new direction for understanding the evolution of castes. Genetics Research International.

[ref-63] Welch M, Lister R (2014). Epigenomics and the control of fate, form and function in social insects. Current Opinion in Insect Science.

[ref-64] Yan H, Bonasio R, Simola DF, Liebig J, Berger SL, Reinberg D (2015). DNA methylation in social insects: how epigenetics can control behavior and longevity. Annual Review of Entomology.

[ref-65] Yan H, Simola DF, Bonasio R, Liebig J, Berger SL, Reinberg D (2014). Eusocial insects as emerging models for behavioural epigenetics. Nature Reviews Genetics.

[ref-66] Zhang Y, Shields T, Crenshaw T, Hao Y, Moulton T, Tycko B (1993). Imprinting of human H19: allele-specific CpG methylation, loss of the active allele in Wilms tumor, and potential for somatic allele switching. American Journal of Human Genetics.

